# Comparison of clinicopathological parameters, prognosis, micro-ecological environment and metabolic function of Gastric Cancer with or without Fusobacterium sp. Infection

**DOI:** 10.7150/jca.50918

**Published:** 2021-01-01

**Authors:** Siru Nie, Ang Wang, Yuan Yuan

**Affiliations:** 1 Tumor Etiology and Screening Department of Cancer Institute and General Surgery, The First Hospital of China Medical University, Shenyang 110001, China.; 2 Key Laboratory of Cancer Etiology and Prevention in Liaoning Education Department, The First Hospital of China Medical University, Shenyang 110001, China.; 3 Key Laboratory of GI Cancer Etiology and Prevention in Liaoning Province, The First Hospital of China Medical University, Shenyang 110001, China.

**Keywords:** Fusobacterium sp., gastric cancer, 16S rRNA, clinicopathological feature, micro-ecological environment, metabolic function

## Abstract

**Background:** Fusobacterium sp. plays a crucial role in the tumorigenesis and development of gastrointestinal tumors. Our research group previously disclosed that Fusobacterium sp. was more abundant in gastric cancer (GC) tissues than adjacent non-cancerous (NC) tissues. However, Fusobacterium sp. did not exist in all GC tissues and the differentiated features of GC with or without Fusobacterium sp. infection is not clear.

**Methods:** The expression data of 61 GC tissues came from 16S rRNA gene sequencing. Comparison groups were defined based on sOTU at the genus level of Fusobacterium sp., which was performed by the Qiime2 microbiome bioinformatics platform. We used Chi-square and Fisher's exact test to compare clinicopathological parameters, and used Kaplan-Meier analysis, Cox univariate and multivariate analysis to compare prognosis. Micro-ecological environment comparison was characterized by 16S rRNA gene sequencing, and the metabolic function prediction was applied by PICRUSt2. Results of microbial diversity, differential enrichment genus and metabolic function in GC with or without Fusobacterium sp. infection was validated with 229 GC tissues downloaded from an independent cohort in ENA database (PRJNA428883).

**Results:** The infection rate of Fusobacterium sp. in 61 GC tissues was 52.46% and elderly GC patients were more prone to Fusobacterium sp. infection. GC patients infected with Fusobacterium sp. were more likely to have tumor-infiltrating lymphocytes and p53 expression. The microbial diversity and microbial structure showed significant differences between two GC tissue groups with 42 differential enrichment genera. The metabolic function of Fusobacterium sp.-positive GC tissues was related to the biosynthesis of lysine, peptidoglycan, and tRNA. The differences in microbial structure, the existence of some differential enrichment genera and the metabolic function of Fusobacterium sp.-positive GC tissues, were then validated by 229 GC tissues of an independent cohort.

**Conclusions:** Fusobacterium sp. infection can affect the phenotypic characteristics, micro-ecological environment, and metabolic functions of GC, which may provide a basis for further exploring the relationship between Fusobacterium sp. infection and carcinogenesis of GC.

## Introduction

Fusobacteria are Gram-negative, obligated anaerobic, rod-shaped bacterium with neither motility nor spore formation, acting as a normal part of the oropharyngeal, gastrointestinal tract, and genital microbiota 1, 2. In recent years, studies have demonstrated that Fusobacterium sp. plays a crucial role in the tumorigenesis and development of gastrointestinal tumors. For example, Fusobacterium sp. DNA can be isolated and detected from colon cancer tissues 3-7 and the level of bacterial DNA correlates with the depth of tumor infiltration tissue in esophageal cancer 7. Our research group previously analyzed mucosa-associated microorganisms from matched gastric cancer (GC) tissues and adjacent non-cancerous (NC) tissues through 16S rRNA gene sequencing and found that the distribution of Fusobacterium sp. in GC and NC tissues was different: Fusobacterium sp. was more abundant in GC tissues than NC 8. After further observation and analysis of the data, we figured out that Fusobacterium sp. did not exist in all GC tissues and the differentiated features of GC with or without Fusobacterium sp. infection is not clear. In previous GC microbial related studies, most of them focused on exploring the diversity of the flora and the construction of ecological networks, but not on the comparison of a particular bacterial genera 9-12. Meanwhile, researches associated with Fusobacterium sp. infection mainly kept an eye on comparing the expression level of Fusobacterium sp. between GC and normal tissues, but did not analyze the effect of Fusobacterium sp. infection. The specific role and mechanism of Fusobacterium sp. in GC have not been reported, either. In the present study, we used Qiime2 microbiome bioinformatics platform to conduct an in-depth analysis of the 16S rRNA sequencing data, further compared clinicopathological parameters, prognosis, micro-ecological environment and metabolic function of GC tissues infected with or without Fusobacterium sp., aiming to provide an experimental basis for exploring Fusobacterium sp. infection and carcinogenesis of GC.

## Materials and methods

### Study population and 16S rRNA gene sequencing

This study consisted of 61 GC patients who received subtotal gastrectomy at the First Affiliated Hospital of China Medical University from June 2012 to June 2014. The recruited 61 GC tissues were immediately frozen and stored at -80 °C after the operation. Genomic DNA was then extracted for amplification and gene sequencing based on V4-V5 regions of 16S rRNA gene after quality examinations. The inclusion and exclusion criteria of patients, the methods of DNA extraction, and 16S rRNA gene sequencing were the same as our previous paper 8. This study has been approved by the Human Ethics Review Committee of The First Hospital of China Medical University and the written informed consents have been obtained from all patients. The original 16S rRNA sequencing data of 229 Chinese GC tissues were downloaded from the European Nucleotide Archive (ENA) database with the bioproject number of PRJNA428883 to validate results of the present study 12.

### Data processing and detection of Fusobacterium sp. infection

After 16S rRNA gene sequencing raw sequence data was split, intercepted, spliced, and filtered, we obtained clean tags (fastq reads data). Fastq reads data was then processed by using Qiime2 microbiome bioinformatics platform 13. OTU denoising was carried out to obtain sOTU data by qiime deblur denoise-16S 14. Taxonomy was assigned to sOTU against Greengene 18.3 database with 99% similarity. The absolute abundance of sOTU at genus level was determined as the criterion of Fusobacterium sp. infection in GC: sOTU value > 0 was regarded as Fusobacterium sp. infection (Fusobacterium sp. positive), while sOTU=0 was regarded as no or very low Fusobacterium sp. infection (Fusobacterium sp.- negative).

### The collection of clinicopathological variables and prognostic information

For the recruited 61 GC patients, general clinicopathological variables information including age, gender, tumor size, differentiation, Lauren's classification, depth of invasion, tumor lymphocyte infiltration, vascular cancer embolus, lymphatic metastasis, and TNM stage were collected from their medical records. The immunohistochemical information of tumor biomarkers including Ki67, p53, CEA, Her-2 were collected from the patients' pathological reports. The prognostic information including survival state, distant metastasis, overall survival (OS), and date of death were collected through telephone follow-up every six months with a follow-up period until November 13, 2019.

### Differential genus enrichment and microbial diversity analysis

R software vegan package was used to calculate α diversity indexes (richness, Chao1, ACE, Shannon, Simpson, phylogenetic diversity (PD) whole diversity indexes) 15. q2‐diversity in Qiime2 was used to calculate β-diversity metrics (weighted UniFrac, unweighted UniFrac, Jaccard distance, and Bray-Curtis dispersion). β-diversity differentiations between Fusobacterium sp. positive and negative GC tissue groups were compared through permutational multivariate analysis of variance (PERMANOVA) 16, 17. Differential genus enrichment between two GC groups was analyzed by linear discrimination analysis effect size (LEfSe). The linear discriminant analysis (LDA) absolute values > 2 and *P* values < 0.5 were considered genus enrichment differentiation statistically significant. Spearman's correlation analysis was performed to analyze the correlation coefficients (r values) between enrichment genera. Genera with strong correlations (r absolute values >0.7) were screened to construct a correlation network visualized by Cytoscape V.3.7.0.

### Metabolic Function prediction of differential enrichment flora

PICRUSt2 was used to predict metabolic functions by analyzing sOTUs in Fusobacterium sp. positive and negative GC tissues based on Kyoto Encyclopedia of Genes and Genomes (KEGG) database and MetaCyc database 18. LEfSe was used to calculate differential KEGG orthology (KO) and metabolic pathways between two GC groups.

### Statistical Analysis

Mann-Whitney U test was used to compare α diversity between Fusobacterium sp. - positive and negative GC tissues. PERMANOVA was used to test β diversity differences. LEfSe was performed to analyze differential genus enrichment and differential functions. LDA absolute values > 2.0 were considered significant. Spearman's correlation analysis was performed to calculate correlation coefficients (r values) between enrichment genera with r absolute values > 0.7 as a strong correlation. Chi-square test, Fisher's exact test, and Mann - Whitney U test were applied to analyze the correlation of Fusobacterium sp. infection and clinicopathological variables. Kaplan-Meier analysis, Cox univariate and multivariate analysis were applied to evaluate the differences in OS between two GC groups. Mann-Whitney U test, Chi-square test, Fisher's exact test, Kaplan Meier analysis, Cox univariate and multivariate analysis were all statistically analyzed by SPSS 25.0 software (SPSS Inc., Chicago, IL, United States). The pictures were drawn by Prism GraphPad 8.4.1, Cytoscape V.3.7.0 and R software. *P <* 0.05 was considered statistically significant.

## Results

### Basic information of the study population and Fusobacterium sp. infection status

**Figure 1:**
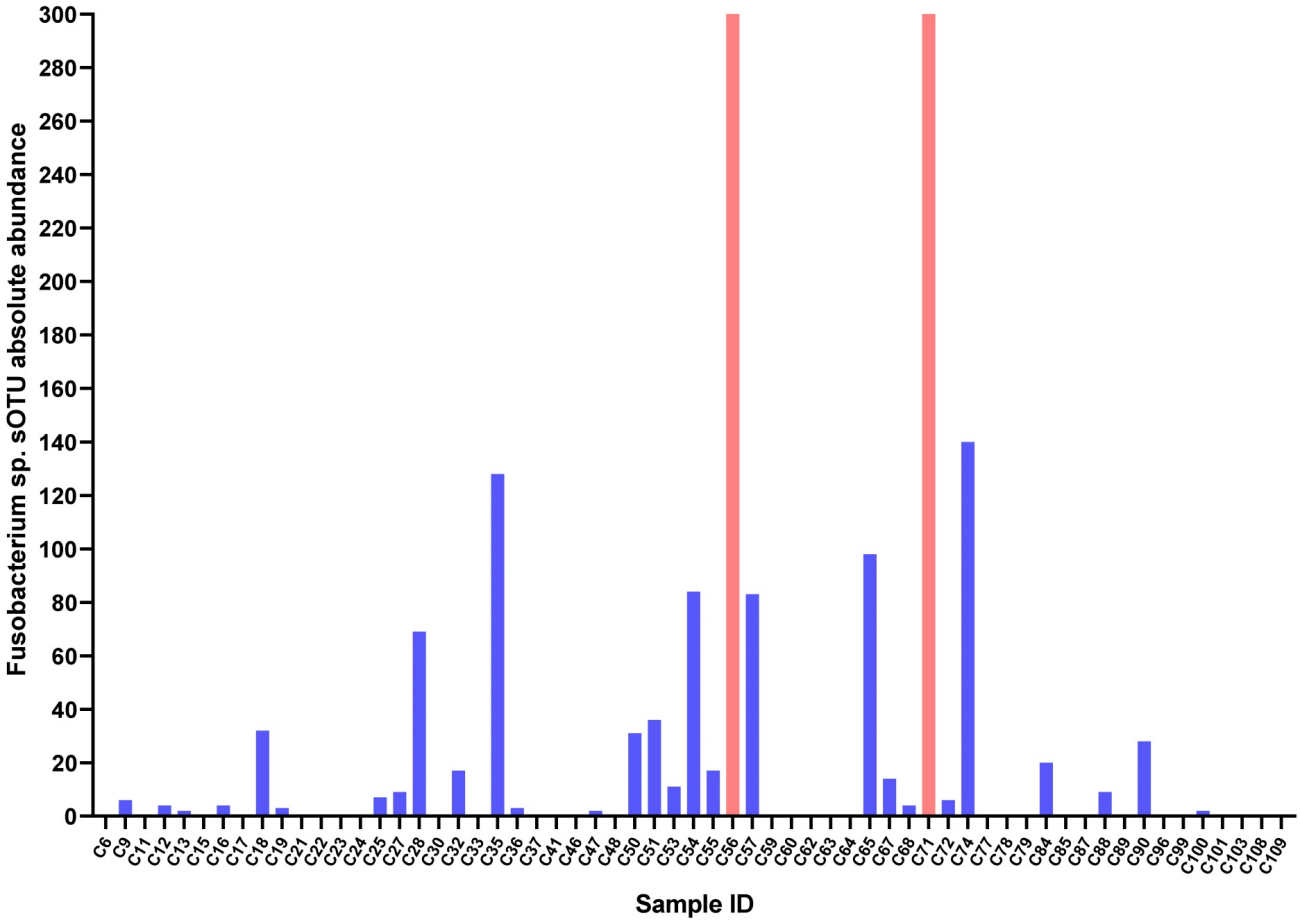
Fusobacterium sp. sOTU absolute abundance in GC tissues. C56 sOTU absolute abundance value is 4311, C56 sOTU absolute abundance value is 397.

In this study, the age of 61 GC patients ranged from 26 to 83 years with a median age of 61 years. Among these patients, 46 were male (75.41%) and 15 were female (24.59%). Through the Qiime2 microbiome bioinformatics platform, we obtained sOTU absolute abundance data (Table S1). Taking absolute abundance of sOTU at genus level as the criterion to determine Fusobacterium sp. infection in GC, we found 30 GC tissues were infected by Fusobacterium sp., while 31 were not. That is, the infection rate of Fusobacterium sp. in GC was 52.46% (Figure 1).

### The differences of clinicopathological parameters between GC with or without Fusobacterium sp. infection

In the present study, we compared age, gender, and clinicopathological parameters between Fusobacterium sp.-positive and negative GC tissues. Elderly GC patients were more prone to Fusobacterium sp. infection (*P =* 0.041) and tumor lymphocyte infiltration was related to Fusobacterium sp. infection (*P =* 0.040). However, for gender, tumor size, differentiation, Lauren's classification, depth of invasion, vascular cancer embolus, lymphatic metastasis, and TNM stage, there were no significant differences between two GC groups (*P >* 0.05) (Table 1). For tumor biomarkers, Fusobacterium sp. infection had no relationship with CEA, Ki67, and Her-2 expression, but significantly associated with p53 expression. Compared with Fusobacterium sp.-negative group, GC patients infected with Fusobacterium sp. were more likely to have p53 expression (*P =* 0.016) and the expression level was higher (Table 1).

### The prognosis of GC with or without Fusobacterium sp. infection

In Kaplan-Meier survival analysis, the OS between Fusobacterium sp. negative and positive groups had no significant difference (*P =* 0.899). Considering the above clinicopathological parameters, Kaplan Meier survival analysis also showed no significant difference between two GC groups (*P >* 0.05). After Cox univariate analysis, parameters with *P <* 0.1 (tumor size, differentiation, depth of invasion, vascular cancer embolus, lymphatic metastasis, TNM stage) were included in multivariate analysis. After adjusting parameters that affect prognosis, Fusobacterium sp. infection could not be regarded as an independent risk factor of prognosis (*P >* 0.05) (Table S2).

### Microbial diversity and differential enrichment genus in GC with or without Fusobacterium sp. infection

**Figure 2:**
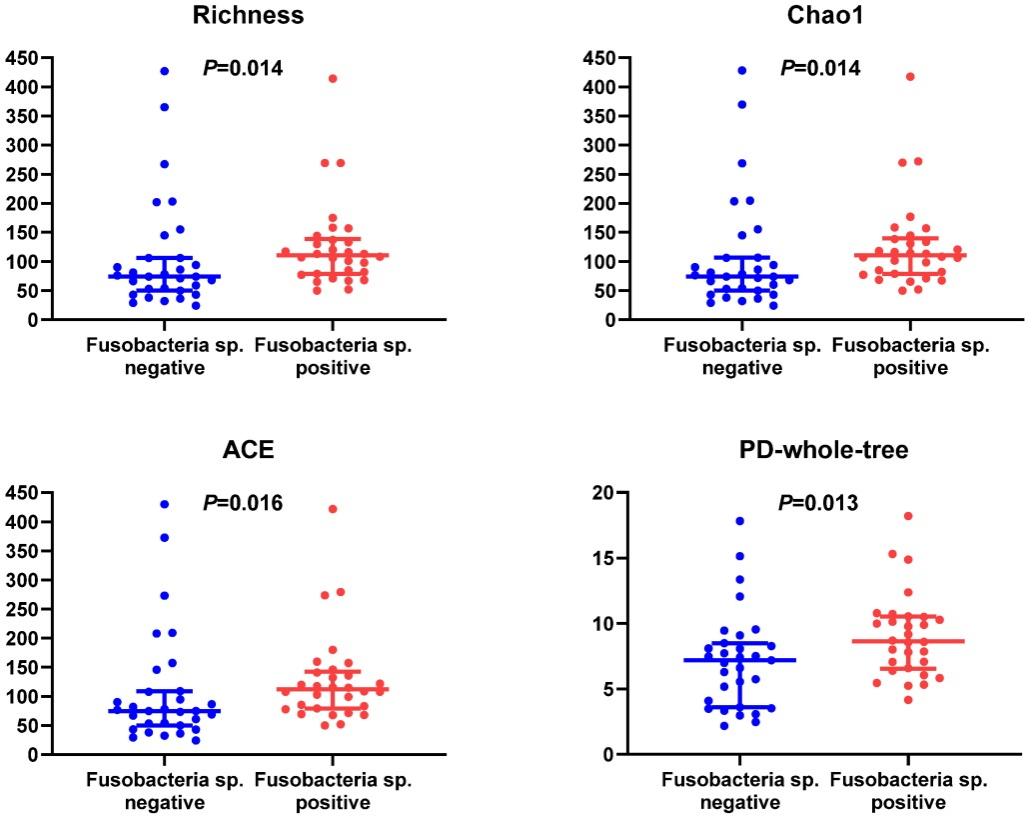
Differences of α diversity indexes (richness, Chao1 index, ACE index) and PD whole tree in GC tissues with or without Fusobacterium sp. Infection.

Microbial diversity between two GC groups had a significant difference. In terms of α diversity, microbial abundance indexes (richness, Chao1 index, ACE index) and phylogenetic diversity (PD) whole tree indexes in Fusobacterium sp.-positive GC tissues were higher than negative ones (Figure 2), while Shannon index, Simpson's index, Pielou, and goods coverage had no statistical difference (*P >* 0.05) (Figure S1). In terms of β diversity, we calculated Bray-Curtis dispersion, Jaccard, unweighted UniFrac, weighted UniFrac distance metrics. Among them, Jaccard (*P =* 0.001), unweighted UniFrac (*P =* 0.001), and weighted UniFrac distance metrics (*P =* 0.042) showed a statistical difference between two GC groups (Table S3), suggesting that Fusobacterium sp. infection status in GC could affect the structure of microbiome.

**Figure 3:**
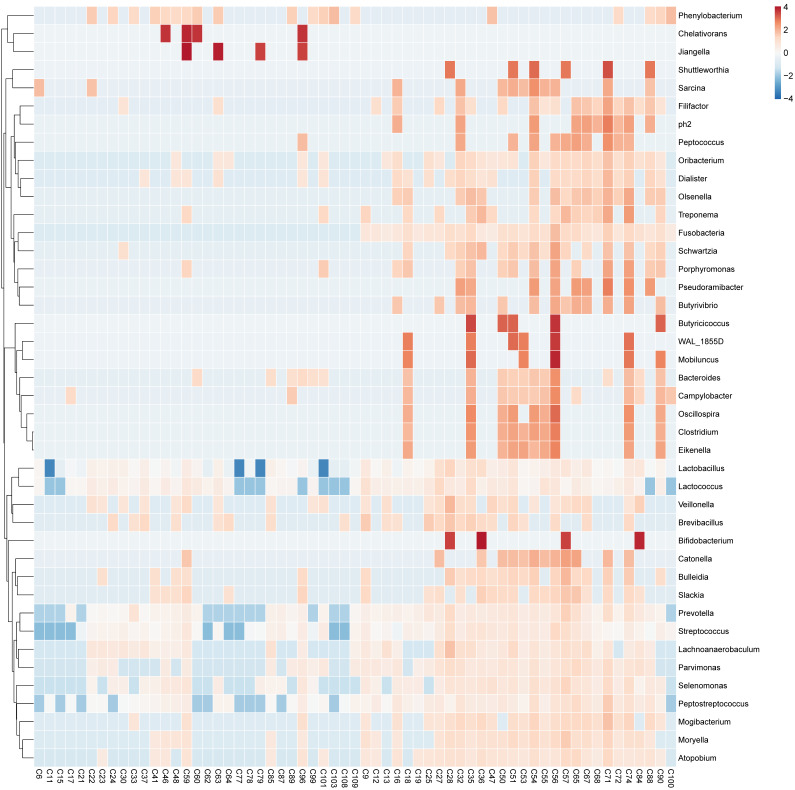
Differential enrichment genus in GC tissues with or without Fusobacterium sp. Infection.

**Figure 4:**
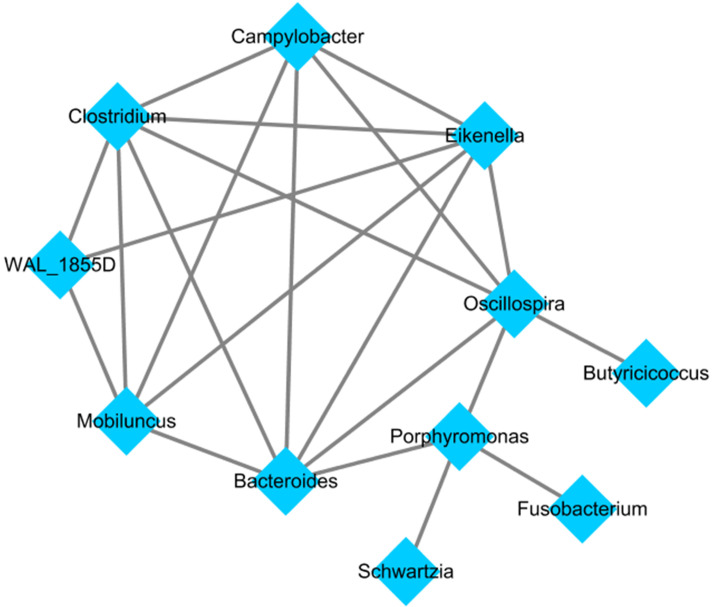
Bacteria interaction network in Fusobacterium sp. - positive GC tissue.

After applying the LEfSe algorithm, we screened differential enrichment genera between Fusobacterium sp. positive and negative GC groups and construct a microbial interaction network. Among all the 42 differential genera obtained, Jiangella、Phenylobacterium and Chelativorans had higher abundances in Fusobacterium sp. - negative GC tissues, while 39 genera (such as Prevotella, Bacteroides and Pepsostretococcus, etc.) had higher abundances in Fusobacterium sp. - positive ones (Figure 3). Bacteria interaction network was constructed by Spearman correlation analysis based on the strong correlation genera (r > 0.7) (Figure 4). In Fusobacterium sp. - positive GC tissues, it showed more correlations among bacteria with Fusobacterium sp. strongly correlating with Porphyromonas sp. (*r=* 0.72, *P <* 0.05). While in the Fusobacterium sp. - negative group, the correlation was more simple (Table S4).

### Functional analysis of metabolic pathways in GC with or without Fusobacterium sp. infection

We used PICRUSt2 to predict metabolic function and found some enzymes and metabolic pathways between Fusobacterium sp. positive and negative GC groups were significantly different (*P <* 0.05). Among them, 25 enzymes and 4 KOs were highly enriched in Fusobacterium sp. -positive GC tissues, 14 enzymes and 1 KO were highly enriched in Fusobacterium sp. -negative GC tissues (Figure S2). As for differential metabolic pathways, KEGG and metacyc database were applied to annotate metabolic pathways. In metacyc database, 44 metabolic pathways were highly enriched in Fusobacterium sp. - positive GC tissues and 15 were highly enriched in the negative ones (Figure S2). Among them, the metabolic pathway of L-glutamate degradation V (via hydroxyglutarate) was confirmed to be related to Fusobacteria. In KEGG database, 16 metabolic pathways were highly enriched in Fusobacterium sp. - positive GC tissues, mainly associated with genetic information processing (replication, transcription, and repair) and metabolism of vitamins, amino acids, and compounds; 9 pathways were highly enriched in Fusobacterium sp. - negative GC tissues, mainly associated with carbohydrate metabolism, amino acid metabolism, cell movement, and signal transduction. In both the KEGG and MetaCyc database, three differential metabolic pathways were found and listed in Table 2.

### The validation of microbial diversity, differential enrichment genus and metabolic function in GC with or without Fusobacterium sp. infection with a Chinese independent cohort

To validate the results of the present study, we used a Chinese independent cohort of 229 GC 16S rRNA sequencing data to analyse microbial diversity, differential enrichment genus and metabolic function in GC tissues with or without Fusobacterium sp. infection. As the methods and the determination criterion of Fusobacterium sp. described above, sOTU was obtained through Qiime2 microbiome bioinformatics platform. Of the 229 GC patients, 75 were infected by Fusobacterium sp., while 154 were not, with a 32.75% infection rate of Fusobacterium sp. In the analysis of microbial diversity, α diversity showed no statistical differences (Figure S3) between two GC Fusobacterium sp. infection groups, while β diversity index Unweighted Unifrac distance metrics (*P =*0.039) and Weighted Unifrac distance metrics (*P =*0.023) were statistically significant. The consistent result of β diversity index indicated that Fusobacterium sp. infection could affect the microbial structure of GC tissues. As for differential enrichment genus, 21 were enriched in Fusobacterium sp.- negative GC tissues and 26 were enriched in the positive ones. Of the 26 genus in Fusobacterium sp.- positive GC tissues, 16 showed the same enrichment as the above results (Figure S4). However, the correlation of differential genus showed no strong correlation (*r <* 0.7). In Fusobacterium sp.- positive GC tissues, Fusobacterium sp. showed a middle correlation with Catonella sp. (*r=*0.47) and Selenomonas sp. (*r=*0.46). Regarding of the metabolic function, we used PICRUSt2 to predict metabolic pathways through KEGG and metacyc database (Table S5). We figured out that the pathways listed in Table 2 were all included in metabolic pathway predictions in these 229 GC tissues. In general, after using a 229 GC independent cohort to validate the results of microbial diversity, differential enrichment genus and metabolic function in 61 GC tissues with or without Fusobacterium sp. infection, Fusobacterium sp. infection had an impact on microbial structure diversity, flora distribution, genus interaction and metabolic functions (lysine, peptidoglycan and tRNA charging.) in GC tissues. Unfortunately, due to the lack of public information on clinicopathological parameters of these 229 GC patients, the effect of Fusobacterium sp. infection on clinicopathological parameters and prognosis of GC still need further validation and exploration.

## Discussion

In the present study, using the Qiime2 microbiome bioinformatics platform, microbial related analysis, correlation analysis and functional prediction software, we compared clinicopathological parameters, prognosis, micro-ecological environment and metabolic function of GC tissues with or without Fusobacterium sp. infection. The results showed Fusobacterium sp. infection could be detected in GC tissues and was prone to elderly patients. Meanwhile, Fusobacterium sp. infection was positively related to p53 expression and tumor infiltration lymphocytes. In GC tissues, microbial diversity, differential enrichment genus, and metabolic function all showed significant differences between two GC tissue groups. These results may provide a scientific basis for elucidating the impacts of Fusobacterium sp. infection on GC phenotype and micro-ecological environment.

### Fusobacterium sp. could be detected in GC tissues and were age-related

Our previous study has found that the abundance of Fusobacterium sp. in GC tissues is higher than NC tissues 8. Other studies also suggested the abundance differences of Clostridium and Fusobacterium between GC and adjacent NC tissues and the combined detection of Clostridium and Fusobacterium may be used as a bacterial marker for diagnosing GC with 100% sensitivity and 70% specificity 19. Our present study further found that the abundance of Fusobacterium sp. not only differed between GC and NC tissues but also among each GC tissues. Based on the 16S rRNA sequencing, Fusobacterium sp. detection rate of 61 GC tissues was 52.46%, which indicated that over 50% of GC was related to Fusobacterium sp. infection. However, in the 229 GC tissues, Fusobacterium sp. detection rate was only 32.75%. In a previous small sample study of Taiwan in China, Fusobacterium sp. could be detected in 7 out of 11 GC patients, with a 63.64% infection rate 19. Though the detection rate varied in GC tissues, we could still speculated that Fusobacterium sp. infection may not only have a close relationship with the carcinogenesis of GC but also play an important role in the progress of GC. This inference is worthy of in-depth study and further confirmation. Besides, our results showed that the status of Fusobacterium sp. infection had no statistical relationships with gender, but elderly GC patients were more prone to infect Fusobacterium sp. This finding may provide valuable clues for developing age-based precision prevention and for observing differences in various population distributions caused by Fusobacterium sp. infection.

### Fusobacterium sp. infection is related to tumor-infiltrating lymphocytes in GC tissues

In the present study, we compared clinicopathological parameters (tumor size, differentiation, Lauren's classification, depth of invasion, tumor-infiltrating lymphocytes, vascular cancer embolus, lymphatic metastasis, and TNM stage) between GC tissues with or without Fusobacterium sp. infection. A relationship only occurred in tumor-infiltrating lymphocytes: the lymphocyte infiltration was lower in Fusobacterium sp.-negative GC tissues, but higher in Fusobacterium sp.-positive ones. Previous studies on colorectal cancer have demonstrated the relationship between Fusobacterium sp. infection and tumor-infiltrating lymphocytes (especially CD4 ^+^ T cells), which could be affected by MSI status 20-22. In the local immune microenvironment, Fusobacterium sp. may exert a regulatory role in suppressing adaptive immunity by inhibiting the cytotoxicity of NK cells and the reactivity of T cells 23, 24. From here we speculate that under the infection of Fusobacterium sp., the number of infiltrating lymphocytes in GC immune microenvironment will increase in response to pathogens, but the reactivity and anti-tumor adaptive immunity may be suppressed to some extent. To better prevent and treat microbial infection-related tumors, the relationship between Fusobacterium and immune cells still needs further confirmation by *in vivo* and *in vitro* experiments.

### Fusobacterium sp. infection is related to p53 expression in GC tissues

By analyzing Fusobacterium sp. infection and the expression of tumor biomarkers (Ki67, p53, CEA, Her-2), p53 expression was related to Fusobacterium sp. infection: the positive and high expression of p53 was more likely to appear in GC tissues with Fusobacterium sp. infection. Wild-type p53 has the effect of blocking cell cycle and inhibiting tumor progression, and mutant p53 plays a role in promoting cancer 25, 26. Here, due to the short half-life of wild-type p53, the p53 expression in immunohistochemistry was all mutant p53. Previous studies have reported that Fusobacterium sp. could promote the expression of wild-type p53 in oral inflammation and tongue squamous cell carcinoma 27, 28. Other studies on colorectal cancer showed that mutant p53 could express in cancer tissues no matter what infection status of Fusobacterium sp. 29, 30, and the Fusobacterium sp. may promote carcinogenesis by changing APC-K-ras-DCC-p53 in stages 31. There have been few studies analyzing Fusobacterium sp. infection in GC, our study first found out that Fusobacterium sp. had a positive correlation with the expression of mutant p53, which may play a significant role in promoting carcinogenesis. Ki67, acting as a biomarker reflecting tumor proliferation and malignancy, was not related to Fusobacterium sp. infection in our study. Nevertheless, some studies confirmed the effect of Fusobacterium sp. on promoting cancer cell proliferation and invasion, protecting tumors from the attack of immune cells and accelerating cancer progression 32-37. Whether Fusobacterium sp. also plays an important role in the carcinogenesis and development of GC and its mechanism is required to be further explored. In the present study, Her-2, an important index in targeted therapy of GC, was not associated with Fusobacterium sp. infection. The reason for this result may not exclude the restriction of samples and the influence of individual differences, etc. Given the important role of Her-2 in clinical treatment, an in-depth investigation is needed.

### Fusobacterium sp. infection and prognosis of GC patients

In the aspect of prognosis evaluation, our work showed no correlation between Fusobacterium sp. infection and prognosis of GC patients. Previous studies in colorectal cancer and esophageal cancer have shown that Fusobacterium may be used as a prognostic biomarker: the higher of Fusobacterium nucleatum DNA (a species of Fusobacterium sp.), the shorter of OS of cancer patients 38, 39. As a limitation of sample size, individual differences, other factors that affect the status of Fusobacterium sp. infection and the initial stage of Fusobacterium sp. research on GC, the effect of Fusobacterium sp. infection on the prognosis of GC needs further confirmation.

### Differential enrichment genus and metabolic function analysis in GC tissues with or without Fusobacterium sp. infection

In this study, there were differences in microbial diversity, enriched genera, and metabolic function between GC with or without Fusobacterium sp. infection. Compared with Fusobacterium sp.-negative GC tissues, microbial abundance and community genetic diversity were higher in Fusobacterium sp.-positive group. Differences also occurred in microbial structure, which were further validated by 229 GC tissues. These results indicated that the abundance of Fusobacterium sp.- correlated bacteria may increase due to its infection, causing the overall community to change during the evolution of bacteria and affecting the structure of microbial community, but the specific role still needs to be explored. In further analysis of differential enrichment genus, Phenylobacterium and other two genera had higher abundance in Fusobacterium sp.- negative GC tissues, and 39 genera of Prevotella and Bacteroides had higher abundance in Fusobacterium sp.- positive ones. Among them, Fusobacterium sp. had a strong positive correlation with Porphyromonas. This relationship has ever been confirmed in *H.pylori*-associated superficial gastritis, atrophic gastritis, and intestinal metaplasia 40. Meanwhile, evidences have shown that Porphyromonas can exist in normal gastric mucosa 19, the saliva of gastrointestinal tumor patients 41, and is also involved in the occurrence of esophageal squamous cell carcinoma and colorectal cancer together with Fusobacterium sp. 42, 43. In the validating process of the result, we found 16 genera showed the same enrichment trend. However, neither of them had a strong correlation with each other genus. We speculate that in GC, Fusobacterium sp., through the joint action with Porphyromonas, can affect the distribution of gastric flora without the exclusion of other flora. But the correlation may change due to microbial differences in geographical populations.

After further metabolic function prediction by PICRUSt2, we identified the role of Fusobacterium sp. in metabolic functions of GC tissues. Based on the MetaCyc database, the metabolic pathway of L-glutamate degradation V (via hydroxyglutarate) was confirmed to be related to Fusobacteria in Fusobacterium sp.-positive GC, which may due to the suitable anaerobic environment for the growth of bacteria. Based on the KEGG database, the differences in metabolic functions between two GC groups were mainly related to amino acids, carbohydrate, vitamins, some compounds and energy metabolism, as well as some genetic information processing pathways such as DNA replication, nucleotide excision repair processes and translation processes (ribosome metabolism and tRNA biosynthesis). To improve the accuracy of metabolic function prediction, we integrated the results of these two databases and concluded that the metabolic functions of Fusobacterium sp.-positive GC were related to the biosynthesis of lysine, peptidoglycan and tRNA. This result was fully validated in the independent cohort of 229 Chinese GC tissues. This suggested that Fusobacterium sp. and its related flora may be involved in the biosynthesis of lysine secreted by gastric juice and also in the peptidoglycans synthesis of Gram-positive and Gram-negative bacterial cell walls. Interestingly, previous studies indicated that the trmFO gene in Firmicutes, Proteobacteria, Fusobacteria, Acidobacteria and an actinomycete could encode the enzyme that catalyzes tRNA formation 44, among which the first three bacteria could also help regulate tRNA to maintain the fidelity of translation 45. This is somewhat consistent with the composition of the differential genus in Fusobacterium sp.-positive GC tissues in our study and also explains the correlation between tRNA biosynthesis and Fusobacterium sp. infection in GC. Our work provided insights into the effect of Fusobacterium sp. infection on the composition of GC microbial community. Furthermore, the differential metabolic pathways could be used as metabolic markers of Fusobacterium sp. associated GC, laying the foundation for further in-depth exploring the effect of Fusobacterium sp. on the metabolic function of GC.

## Conclusion

According to the results of the present study, the infection rate of Fusobacterium sp. was 52.46% in 61 GC tissues. Elderly GC patients were more prone to Fusobacterium sp. infection and the degree of tumor-infiltrating lymphocyte in Fusobacterium-positive GC tissues was higher. GC patients infected with Fusobacterium sp. were more likely to have p53 expression and the expression level was higher. The microbial diversity and microbial structure showed significant differences in GC tissues with or without Fusobacterium sp. infection with 42 differential enrichment genera. Among them, the strong correlation between Fusobacterium sp. and Porphyromonas sp. in Fusobacterium sp. - positive GC tissues suggested that Fusobacterium sp. may affect microbial distribution by affecting Porphyromonas sp. or both. Moreover, the metabolic function of Fusobacterium sp.-positive GC tissues was related to the biosynthesis of lysine, peptidoglycan, and tRNA. Microbial structure diversity, differential enrichment genus and metabolic function were further validated by an independent cohort of 229 Chinese GC tissues. In conclusion, Fusobacterium sp. infection can affect the phenotypic characteristics, micro-ecological environment, and metabolic functions of GC. This may provide a basis and clue for further exploring the relationship between Fusobacterium sp. infection and carcinogenesis, development and prognosis of GC. This may also lay a foundation for further in-depth exploration of the mechanism of Fusobacterium sp. affecting metabolic functions of GC.

## Supplementary Material

Supplementary figures and tables.

## Figures and Tables

**Table 1:** Fusobacterium sp. infection and clinicopathological parameters in GC

Parameters	Fusobacterium sp.-positive	N	Fusobacterium sp.-Negative	N	*P*
Age	65 (53.00-69.26)	30	59.00 (50.00-64.00)	31	*0.041
**Gender**		30		31	0.119
Male	20 (66.67%)		26 (83.87%)		
Female	10 (33.33%)		5 (16.13%)		
**Tumor size**		30		31	0.716
≤6 cm	18 (60%)		20 (64.5%)		
>6 cm	12 (40%)		11 (35.5%)		
**Differentiation**		30		31	0.348
Low and others	26 (86.7%)		24 (77.4%)		
High-middle	4 (13.3%)		7 (22.6%)		
**Lauren's classification**		30		31	0.699
Intestinal	14 (46.7%)		16 (51.6%)		
Diffuse	16 (53.3%)		15 (48.4%)		
**Depth of invasion**		30		31	0.731
T1+T2	25 (48.1%)		27 (51.9%)		
T3+T4	5 (55.6%)		4 (44.4%)		
**Tumor lymphocyte infiltration**		30		31	*0.040
+	10 (33.3%)		11 (35.5%)		
++	6 (20.0%)		14 (45.2%)		
+++	14 (46.7%)		6 (19.4%)		
**Vascular cancer embolus**		30		31	0.372
Negative	17 (56.7%)		21 (67.7%)		
Positive	13 (43.3%)		10 (32.3%)		
**Lymphatic metastasis**		30		31	0.860
Negative	10 (33.3%)		11 (35.5%)		
Positive	20 (66.7%)		20 (64.5%)		
**TNM stage**		30		31	0.878
I-II	12 (40%)		13 (41.9%)		
III-IV	18 (60%)		18 (58.1%)		
**Ki67**		30		31	0.648
≤70%	9 (30%)		11 (35.5%)		
>70%	21 (70%)		20 (64.5%)		
**P53**		30		31	*0.016
Negative	5 (16.7%)		14 (45.2%)		
Positive	25 (83.3%)		17 (54.8%)		
**CEA**		30		31	0.181
(+)	12 (40%)		17 (54.8%)		
(++)	3 (10%)		4 (12.9%)		
(+++)	15 (50%)		10 (32.3%)		
**Her-2**		30		31	0.900
Negative	15 (50.8%)		16 (54.6%)		
Positive	15 (49.2%)		15 (48.4%)		

**Table 2:** Highly enriched metabolic pathways in Fusobacterium sp. - positive GC tissues

KEGG Database	Pathway	MetaCyc Database
ko00300	Lysine biosynthesis	PWY-2941
ko00550	Peptidoglycan biosynthesis	PWY-6470
		PEPTIDOGLYCANSYN-PWY
		PWY-6385
		PWY-6471
ko00970	Aminoacyl-tRNA biosynthesis/tRNA charging	TRNA-CHARGING-PWY
