# Supplementary material for: Comparison of clinicopathological parameters, prognosis, micro-ecological environment and metabolic function of Gastric Cancer with or without Fusobacterium sp. Infection
Source: J Cancer. 2021 Jan 1;12(4):1023–32. doi: 10.7150/jca.50918 (PMC7797643; doi:10.7150/jca.50918)
Supplement: Supplementary file 1 — Supplementary figures and tables. [file jcav12p1023s1.zip › Supplementary materials/Supplementary materials.pdf]

Supplementary Materials

Supplementary Figures and Tables

Table S1. sOTU data in genus level of 61 GC tissues.

Shown in Table S1.xls

Table S2. Prognostic analysis of GC with or without Fusobacterium sp. infection.

Shown in Table S2.xls

**Parameters**	**Univariate analysis**	
	**HR (95%CI)**	**P**
Age (>60/≤60)	1.708 (0.798-3.653)	0.168
Gender (male/female)	0.660 (0.280-1.556)	0.343
Tumor size (>6cm/≤6cm)	2.694 (1.265-5.737)	*0.010
Differentiation (high-middle/low and others)	0.253 (0.060-1.070)	0.062
Lauren's classification (intestinal/diffuse)	1.336 (0.635-2.812)	0.445
Depth of invasion (T1+T2/T3+T4)	0.033 (0.001-1.641)	0.087
Tumor lymphocyte infiltration (+/++/+++)	0.862 (0.554-1.343)	0.512
Vascular cancer embolus (negative/positive)	1.991 (0.946-4.192)	0.070
Lymphatic metastasis (negative/positive)	0.081 (0.019-0.345)	*0.001
TNM stage (Ⅰ-Ⅱ/Ⅲ-Ⅳ)	0.091 (0.027-0.305)	*0.000
Fusobacterium sp. Infection (positive/negative)	0.954 (0.454-2.002)	0.901
Ki67 (>70%/≤70%)	0.719 (0.325-1.591)	0.415
P53 (positive/negative)	1.375 (0.605-3.125)	0.447
CEA (+++/++/+)	0.806 (0.384-1.691)	0.568
C-erb-2 (positive/negative)	0.838 (0.517-1.358)	0.472

**Parameters**	**Multivariate analysis**	
	**HR (95%CI) Fusobacterium sp. Infection (positive/negative)**	**P**
Tumor size		
≤6cm	0.954 (0.285-3.197)	0.939
>6cm	0.429 (0.102-1.798)	0.247
Differentiation		
Low and others	0.660 (0.277-1.571)	0.348
High-middle	0.618 (0.031-12.404)	0.753

Depth of invasion		
T1+T2	NA	NA
T3+T4	0.732 (0.316-1.692)	0.465
Vascular cancer embolus		
Negative	1.161 (0.375-3.592)	0.795
Positive	0.415 (0.109-1.589)	0.199
Lymphatic metastasis		
Negative	0.000 (0.000-1.804E+15)	0.553
Positive	0.895 (0.369-2.174)	0.807
TNM stage		
Ⅰ-Ⅱ	0.313 (0.011-8.703)	0.493
III-IV	0.771 (0.317-1.876)	0.567

Table S3. Differences of β diversity indexes in GC with or without Fusobacterium sp. infection.

β diversity index	PERMANOVA results	
	test statistic	p-value
Bray-curtis	0.700	0.616
Jaccard	3.185	*0.001
Unweighted Unifrac distance metrics	6.538	*0.001
Weighted Unifrac distance metrics	2.463	*0.042

Table S4. Correlations of differential enrichment genera in Fusobacterium sp. - positive and Fusobacterium sp. - negative GC tissues (P<0.05).

Shown in Table S4.xls

Table S5. Metabolic function prediction of metabolic pathways in 229 GC tissues with or without Fusobacterium sp. infection(P<0.05)

Shown in Table S5.xls

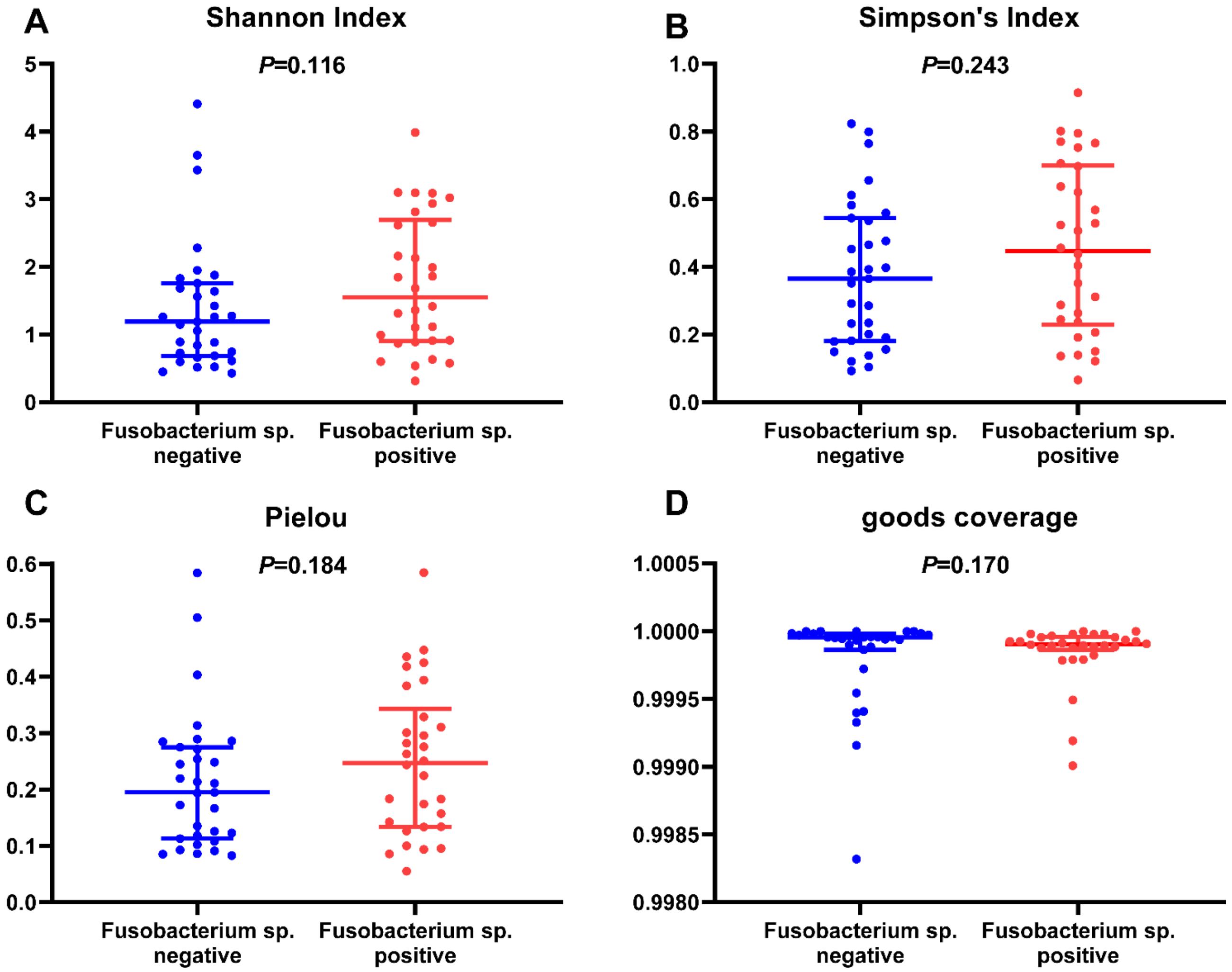

Figure S1. Differences of diversity indexes in GC tissues with or without Fusobacterium sp. infection. (A) Shannon index, (B) Simpson's index, (C) Pielou and (D) goods coverage.

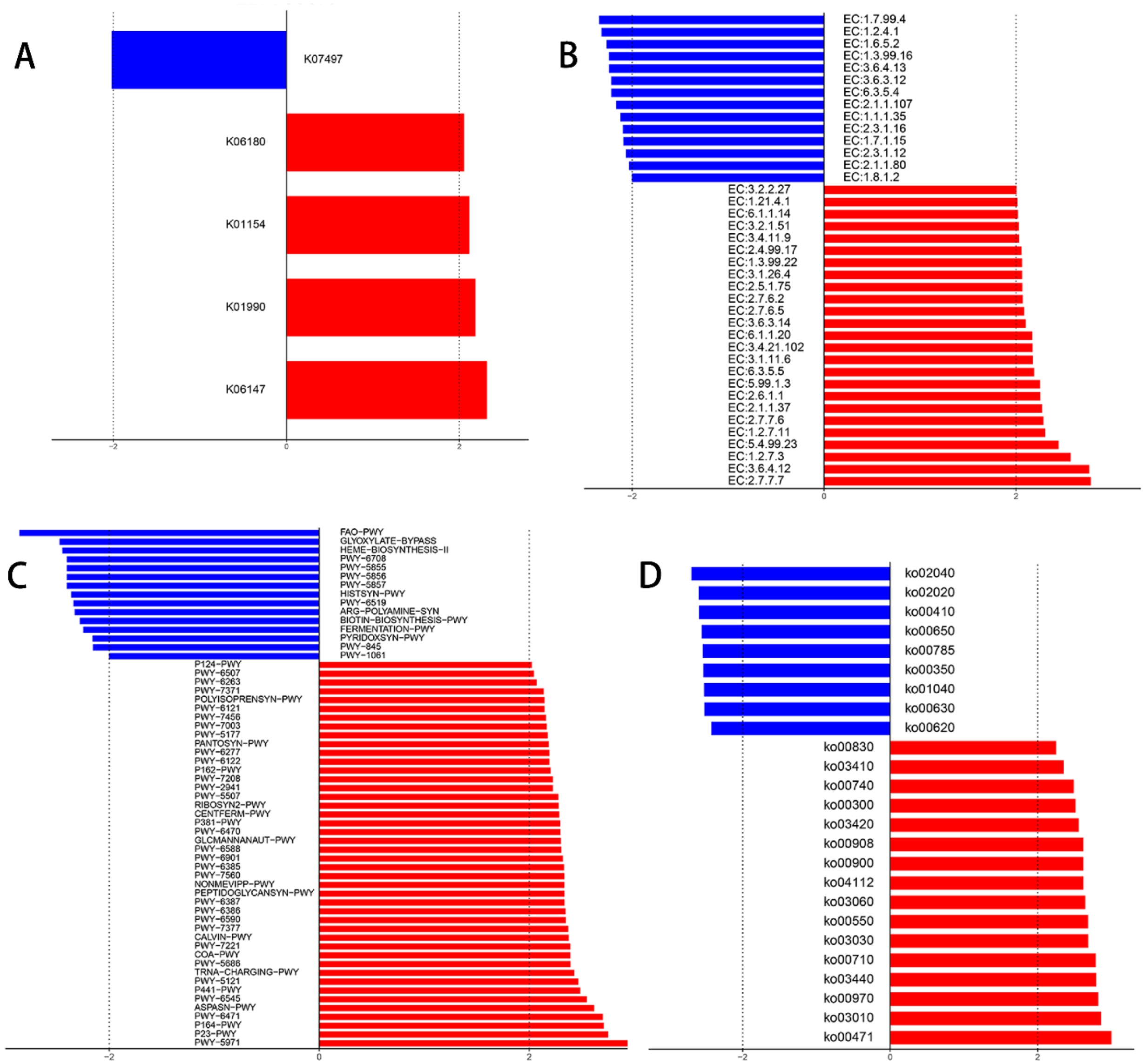

Figure S2. Metabolic function prediction of enzymes, KOs and metabolic pathways in GC tissues with or without Fusobacterium sp. infection. (A) KO differentiation, (B) EC differentiation, (C) pathways differentiation in metacyc database, and (D) pathways differentiation in KEGG database.

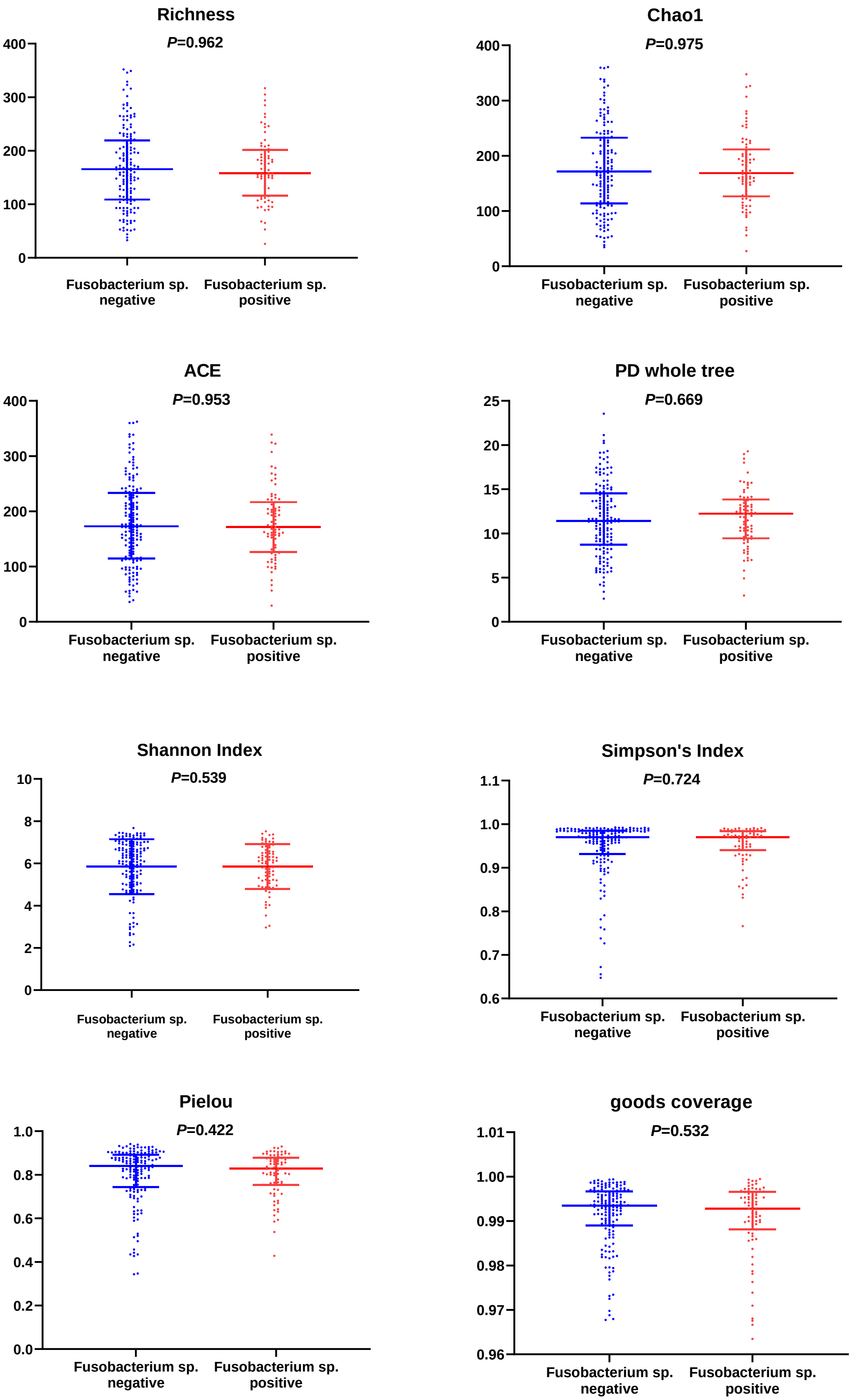

Figure S3: Differences of α diversity indexes in 229 GC tissues with or without Fusobacterium sp. infection

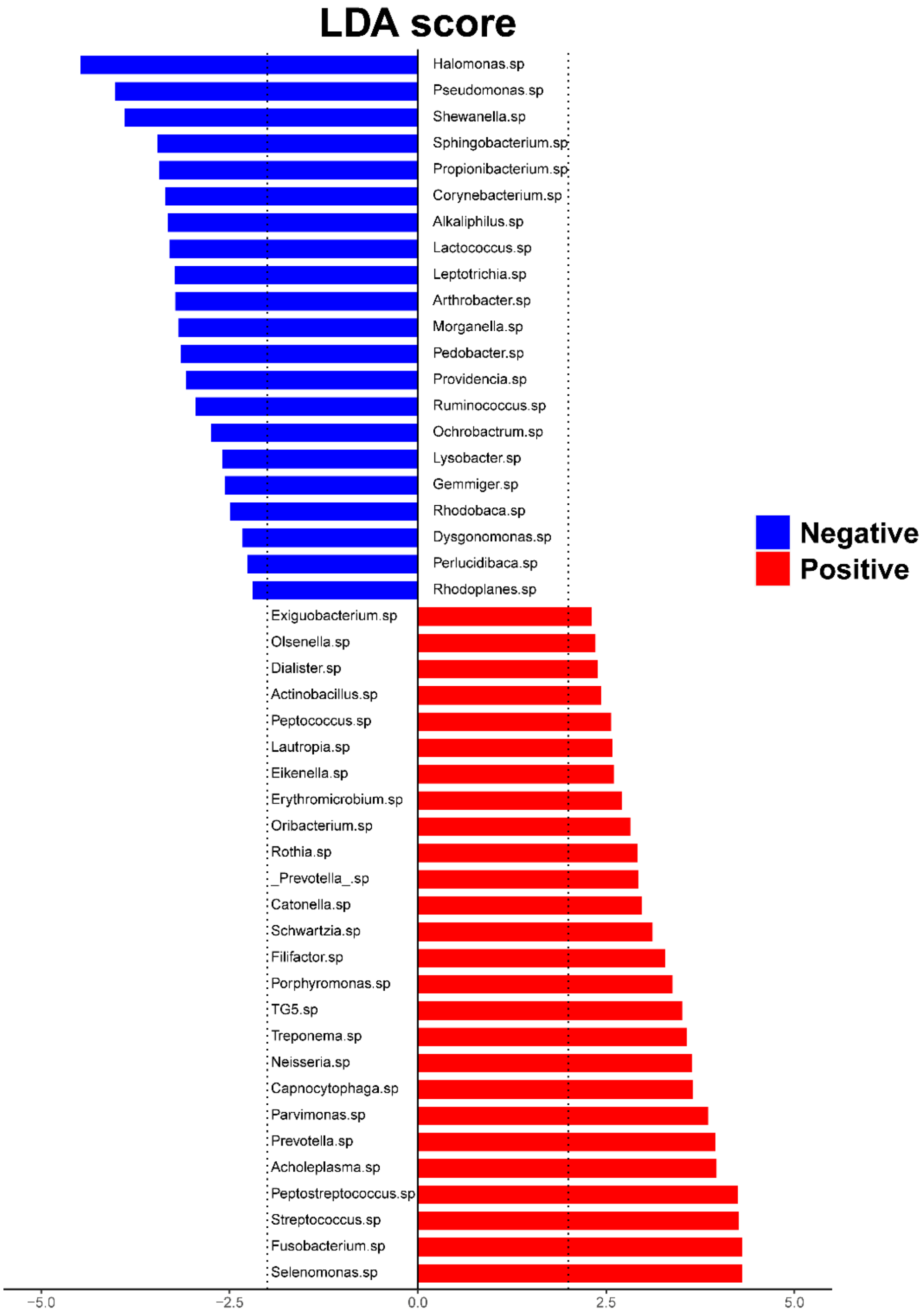

Figure S4: Differential enrichment genus in 229 GC tissues with or without Fusobacterium sp. infection
